# PRELP has prognostic value and regulates cell proliferation and migration in hepatocellular carcinoma

**DOI:** 10.7150/jca.46309

**Published:** 2020-09-09

**Authors:** Runqi Hong, Jiawei Gu, Gengming Niu, Zhiqing Hu, Xiaotian Zhang, Tao Song, Shanliang Han, Liang Hong, Chongwei Ke

**Affiliations:** Department of General Surgery, The Fifth People's Hospital of Shanghai, Fudan University, Shanghai, 200240, P.R. China.

**Keywords:** PRELP, hepatocellular carcinoma, proliferation, prognosis, migration, integrin

## Abstract

**Purpose:** Hepatocellular carcinoma (HCC) is an aggressive and prevalent tumor threatening human health. A previous study suggested low PRELP (proline/arginine-rich end leucine-rich repeat protein) expression was associated with poor patient survival in pancreatic ductal adenocarcinoma (PDAC). However, the role of PRELP in HCC has not yet been illuminated.

**Methods:** PRELP expression analyses were carried out using transcriptomic datasets from the Integrative Molecular Database of Hepatocellular Carcinoma (HCCDB). The correlations between PRELP expression and clinicopathological features, and prognostic analyses were performed with a tissue microarray (TMA) and immunohistochemistry (IHC). The endogenous expression and *in vitro* roles of PRELP were investigated in cultured HCC cell lines. The potential mechanisms were characterized by a Gene Set Enrichment Analysis (GSEA) and gene-gene correlation analyses.

**Results:** We found that PRELP mRNA expression was dramatically decreased in HCCs in comparison with that in adjacent normal tissues (NTs) or hepatic cirrhosis. IHC staining showed that PRELP was down-regulated in HCCs, which mainly located in cytoplasm, and was also found in nuclei. The correlation analyses revealed that PRELP expression was relevant to later p-stages (*p*= 0.028) and tumor size (*p*= 0.001). The overall survival (OS) and relapse free survival (RFS) time was shorter in HCC patients with lower PRELP expression levels than that with higher PRELP expression levels. Overexpression of PRELP inhibited, while knockdown of PRELP promoted proliferation and migration of HCC cells. For potential mechanisms, PRELP may inhibit progression of HCCs by interacting with integrin family members and the extracellular microenvironment.

**Conclusion:** Our findings demonstrated that overexpression of PRELP correlates with better patient survival and inhibits both cell proliferation and migration in HCC. Therefore, PRELP can serve as a potential prognostic biomarker and therapeutic target which deserves further investigation.

## Introduction

Hepatocellular carcinoma (HCC) comprises 75%-85% of liver cancer cases and is one of the most fatal solid malignancies worldwide, with an extremely low 5‑year survival rate 1. The main causes are chronic infection with hepatic B virus (HBV) and exposure to aflatoxin in China. Although prevention measures and interventions like HBV vaccination, health education and surveillance have been put into practice, a considerable part of patients are diagnosed at later stages where surgical resection is not recommended 2. The survival rate of these people has been slightly improved by applying targeted drugs such as sorafenib or regorafenib, a multikinase inhibitor mainly against tumor angiogenesis 3. It is urgent to better know the molecular mechanisms of this malignancy, which would provide creative and effective therapeutic strategies to ease the burden of HCC.

PRELP (proline/arginine-rich end leucine-rich repeat protein), which is highly abundant in collagen-rich tissues, binds collagen to basement membranes and cartilage 4, 5. PRELP belongs to the class II subfamily of the small leucine-rich proteoglycan (SLRP) family and is also known as MST161, MSTP161, prolargin, prolargin proteoglycan or SLRR2A^6^. The neopeptide, which is released from PRELP, is involved in progression and incidence of osteoarthritis (OA) 7, 8. By modulating the β-catenin/connexin43 pathway, PRELP participates in the formation of new bone by osteoblasts 7. In human congenital immunity processes, PRELP could bind with respiratory tract pathogens and impede their adherence to lung epithelial cells 9. A previous study showed that fibroblast adhesion was enhanced on the basis of the binding between N-terminal of PRELP and integrin 10. Furthermore, peptides corresponding to the N‐terminal heparin binding domain of PRELP act as antimicrobial molecules by binding bacterial surface 11. The physiological roles of PRELP in liver still need to be elucidated, however, data from The Human Protein Atlas (HPA) shows that PRELP is secreted to the extracellular matrix and may anchor basement membranes to the underlying connective tissue 12, suggesting its potential function in maintaining normal cellular structure.

Studies of PRELP in human cancers have been reported previously. In a proteomic study, the authors demonstrated that PRELP overexpression was associated with poor patient survival in resectable pancreatic ductal adenocarcinomas (PDAC) 13. In another study conducted in human brain tumors, the authors documented only limited potential of PRELP as a prognostic predictor 14. Besides, data mining and bioinformatics analyses seemed unable to elicit the role of PRELP in bladder urothelial carcinoma 15. While most of these are bioinformatics studies, the role of PRELP in HCC remains unknown. The Integrative Molecular Database of Hepatocellular Carcinoma (HCCDB), which collected fifteen public HCC expression datasets, is established to analyze the differential expression of genes conveniently and efficiently 16. Hence, we utilized this database to investigate the expression of PRELP in HCC at the beginning.

In this study, we compared PRELP mRNA expression levels between HCCs and adjacent normal tissues (NTs) or hepatic cirrhosis by analyzing several public datasets. We utilized a tissue microarray (TMA) and immunohistochemistry (IHC) to investigate the prognostic value of PRELP in HCCs. We studied the *in vitro* activities of PRELP in cultured HCC cells using proliferation and migration assays. Besides, we explored the potential mechanisms of PRELP based on the Gene Set Enrichment Analysis (GSEA) and gene-gene correlation analyses.

## Materials and Methods

### Data collection

Fifteen transcriptome datasets were accessed from HCCDB 16, which is used to analyze the differential gene expression. These datasets include GSE14520 17, 18, GSE63898 19, GSE54236 20, GSE64041 21, GSE22058 22, GSE112790 23, GSE76427 24, GSE10143 25, GSE36376 26, GSE46444 27, GSE14323 28, GSE25097 29, GSE6764 30, the Liver Hepatocellular Carcinoma Project of the Cancer Genome Atlas (TCGA-LIHC), and the Liver Cancer-RIKEN, JP Project from the International Cancer Genome Consortium (ICGC-LIRI-JP) 31 and were analyzed for PRELP mRNA expression between HCCs and hepatic NTs or cirrhosis.

### Cell lines

A hepatic cell line (L02) and four HCC cell lines (HCCLM3, MHCC97-H, SMMC-7721, Alexander cells) were kindly provided by Dr. Lijie Ma at Zhongshan Hospital, Fudan university (Shanghai, China). HCC cell lines SK-HEP-1 and Hep3B2.1-7 were gifted from Prof. Yongzhong Liu at the Cancer Research Institute, Shanghai Jiao Tong University (Shanghai, China); Two HCC cell lines (HepG2 and PLC/PRF/5) were kindly provided by Prof. Xiuping Liu at Shanghai Cancer Hospital, Fudan university (Shanghai, China). All cell lines used in this study were regularly authenticated by morphological observation and tested for the absence of mycoplasma contamination (MycoAlert; Lonza Rockland, Rockland, ME, USA). All Cell lines were cultured at 37℃ with 5% CO2 in DMEM (Sangon Biotech, Shanghai, China) with 10% fetal bovine serum, 100 µg/mL penicillin, and 100 mg/mL streptomycin in a humidified incubator.

### Short hairpin RNAs (shRNAs) construction and lentiviruses infection

Plasmids with PRELP-targeting shRNAs or a nontargeting scrambled RNA (scramble), and the recombined plasmids of PRELP or vector were synthesized by Shanghai GeneChem Co., Ltd. These plasmids were well packaged into lentiviruses as previously reported 32. Four target shRNA sequences are as following: TTCGGCTTAACTACAACAA (shPRELP1), GTAACAAGATTGAGACCAT (shPRELP2), CTCTTAATTGCTCTAACAA (shPRELP3), and TGGTTTCTTTCAAGTTTAA (shPRELP4). All cells were infected and screened as described previously 33.

### Samples and IHC

A TMA containing 90 cases of HCC patients was prepared by Shanghai Tufei Biotechnology Co., Ltd. The inclusion criteria for eligible patient selection were: Child-Pugh A liver function; histologically confirmed HCC; age >18 years; surgery with radical intent. The exclusion criteria were: tumor size <0.5 cm; simultaneous malignancies from other organs; previous systemic treatment; incomplete clinical data; lost follow-up. The clinicopathological characteristics of these patients are presented in** Table 1.** All slides were dewaxed in xylene and rehydrated in graded ethanol, then pretreatment in fresh 3% H_2_O_2_ to quench endogenous peroxidase activity was performed and washed in double-distilled water 3 times. Subsequently, antigen retrieval with citrate buffer pH 6.0 (1:50 dilution; Beyotime Biotechnology, Shanghai, China) was performed in an autoclave and incubated with goat serum (1:20 dilution; cat. no. C0265; Beyotime Biotechnology, Shanghai, China) at room temperature for 1 h, then slides were incubated with rabbit anti-PRELP polyclonal antibody (1:50 dilution; Absin-bio, Shanghai, China.) at 4°C overnight. After washing with PBS three times, the horseradish peroxidase HRP-conjugated goat anti-rabbit secondary antibody was covered on slides for 1h at room temperature washed with PBS 3 times. Then, these sections were reacted with 3,3′-diaminobenzidine (1:25 dilution; cat. no. GK500705; Gene Tech Co., Ltd.) at room temperature for 5 min and counterstained with 100% hematoxylin (Beyotime Biotechnology, Shanghai, China) at room temperature for 2 min. Finally, slides were washed in running water for 45 min before dehydration and clearing. Slides was scanned by the ultra-compact Aperio CS2 image capture device and processed by Aperio ImageScope (Leica Biosystems, Germany). The intensity of staining (0, negative; 1, weak; 2, moderate; and 3, strong) was multiplied by the percentage of positive tumor cells (0-100%) to generate the modified H-score in a range from 0 to 300. PRELP staining was categorized as high or low expression using the median H-score 33.

### RNA extraction and quantitative real-time polymerase chain reaction (qPCR)

Total RNA was isolated from cell cultures using RNAiso Plus (Takara Bio, Kusatsu, Japan) as to the manufacturer's instructions. A 2^-ΔΔCT^ method was utilized to count the relative expression of target sequences as we described in a previous report 34. The sequences for RT-PCR primers were as following: PRELP forward primer 5'CAGCCAACAAGACGACCAAGA3' and PRELP reverse primer, 5'CAGTCAGGGAAGATAGATGGAGG3'. Glyceraldehyde 3-phosphate dehydrogenase (GAPDH) served as an internal control. Experiments were independently repeated three times, in duplicate.

### Protein extraction and western blotting (WB)

After 48 hours of infection, total protein was extracted from cells using RIPA lysis buffer (Beyotime Biotechnology, Shanghai, China), phenylmethylsulfonyl fluoride (Beyotime Biotechnology, Shanghai, China) and proteinase inhibitor cocktail solution (Roche, Basel, Switzerland). The whole extraction process was performed on ice. Then protein samples were centrifuged for 20 min at the speed of 12000 r/min at 4°C. The protein concentration was quantitated using the bicinchoninic acid protein assay (Beyotime Biotechnology, Shanghai, China) as recommended by the manufacturers. SDS-PAGE loading buffer (Beyotime Biotechnology, Shanghai, China) was add to the supernate at the proportion of 25%, then protein was heated at 95°C for 5 min. Western-blot assays were performed as our previous report 35 using rabbit anti-PRELP polyclonal antibody (1:1,000 dilution; Absin-bio, Shanghai, China). GAPDH (1:2,000 dilutions, rabbit anti-human; Beyotime Biotechnology, Shanghai, China) served as a loading control.

### Proliferation assay

Cell suspensions of SMMC-7721 and HCCLM3 cells infected with corresponding lentiviruses were seeded in 96-well plates for 2 × 10^3^ cells per well and cultured for 24, 48, 72 or 96 h. Then 10 uL Cell Counting Kit-8 (CCK-8) reagent [10% (v/v) in serum-free DMEM; Sangon biotech, Shanghai, China] was added to each well and cultured at 37°C for 1 h. A microplate reader (BioTek Synergy 2; BioTek, Winooski, VT, USA) was used to detect the optical density (OD) at 450 nm.

### Colony formation assays

Infected cells were plated in 12-well plates (1 × 10^2^ cells per well), and cultured for 14 days. The medium was changed every three days then cells were fixed in 4% phosphate-buffered paraformaldehyde and stained by 1% crystal violet when macroscopic colonies appeared in the plate. The plates were rinsed with running water then dried in the air before scanning. The colony number was counted on scanned pictures.

### Transwell migration assay

Infected cells were added to 200 μL serum-free DMEM in the upper chamber of a transwell insert. The lower chamber was filled with the medium containing 20% FBS. After 24 h incubation at 37°C, the cells on the upper side of the membrane were removed with a cotton swab, and those on the lower surface were fixed in 4% phosphate-buffered paraformaldehyde for 20 min and stained with crystal violet (0.1% in PBS) for 15 min. Cells were photographed in six randomly selected fields per well with an inverted microscope and the numbers of migrated cells were counted.

### Scratch wound healing assay

Infected cells (4 × 10^5^ cells/mL) were cultured in 12-well plates until confluence. After 24 h of serum-free cultivation, a cross wound was scratched using a 200 μL pipette tip. After washing twice with PBS, the wound areas were photographed under an inverted microscope at 0 and 24 h. Then the wound areas were compared and analyzed as previously described 35.

### Gene set enrichment analysis

The Gene set enrichment analysis (GSEA) is developed by the Broad Institute website, which is normally used to analysis the distribution of the predefined genes in ranked genes associated with complex phenotypes. After the classification of high or low PRELP expression, we managed to investigate whether there were potential mechanisms of PRELP in HCC progression. The TCGA-LIHC dataset was analyzed using the module of the Kyoto Encyclopedia of Genes and Genomes (KEGG) in GSEA 35.

### Statistical analysis

Statistical analyses were conducted by using the SPSS statistical software for Windows, version 22 (IBM Corp., Armonk, NY, USA), Microsoft Excel 2010 (Microsoft, Redmond, WA, USA) and GraphPad Prism7 (GraphPad, San Diego, CA, USA). An independent-samples *t* test was used to analysis the difference of PRELP mRNA expression between HCCs and NTs or hepatic cirrhosis. Comparisons among multiple groups were performed by one-way analysis of variance. The Bonferroni's post hoc test was performed after pairwise comparisons. The Pearson's χ^2^ test and Fisher's exact test were utilized to analyze the correlation between PRELP protein expression and clinicopathological features. Univariate and multivariate Cox proportional hazards models were used to explore key factors that are significantly associated with patient prognoses. The survival curves were graphed using Kaplan-Meier method and the difference between two curves was analyzed by log-rank test. The p value less than 0.05 was defined as statistically significant. All statistical tests were two-sided.

## Results

### PRELP is down-regulated in HCC tissues

**Figure 1:**
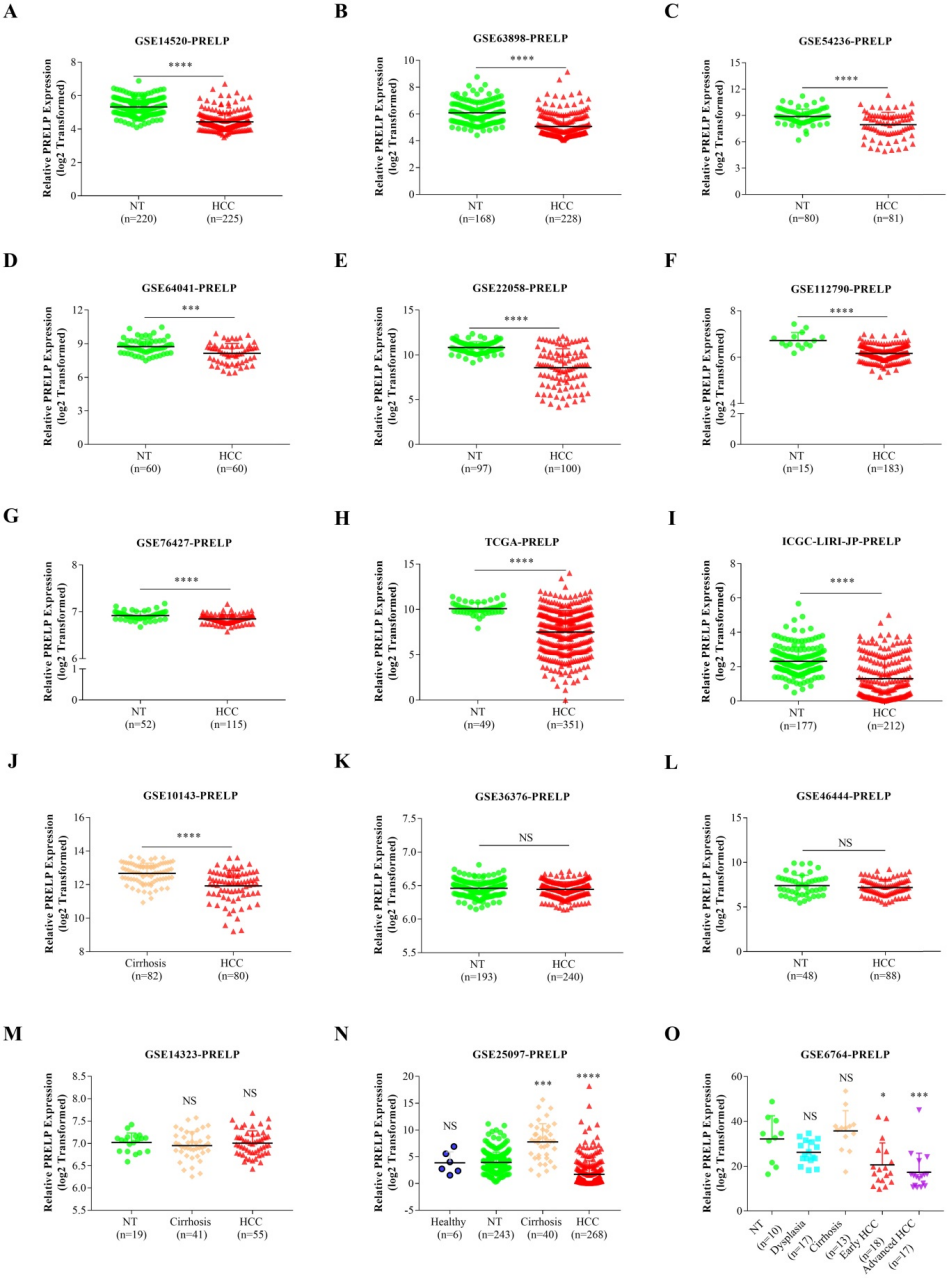
** PRELP was downregulated in HCCs.** The comparisons of PRELP mRNA expression between HCCs and adjacent NTs or hepatic cirrhosis in 12 transcriptomic datasets (**A-L**). The comparison of PRELP mRNA expression among HCCs, adjacent NTs and hepatic cirrhosis (**M**), or together with healthy hepatic tissues (**N**). PRELP mRNA expression levels in different stages during HCC progression (**O**). Abbreviations: HCC: hepatocellular carcinoma; NTs: normal tissues; NS: not significant; *: *p*<0.05; **: *p*<0.01; ***: *p*<0.001; ****: *p*<0.0001.

In order to explore the expression pattern of PRELP mRNA, we conducted a comparison between HCCs and adjacent tissues based on the transcriptomic data acquired from the public database HCCDB. PRELP mRNA expression was significantly down-regulated in HCCs compared with that in adjacent NTs or hepatic cirrhosis in 10 out of the 15 datasets (**Figure 1A-1J**). However, the result showed no significant difference between HCCs and adjacent NTs with regard to PRELP expression in three datasets (**Figure 1K-1M**). Analysis of a dataset which provided healthy liver tissues demonstrated that PRELP expression was dramatically reduced in HCCs compared with that in healthy liver tissues or NTs (**Figure 1N**). In another dataset that listed different disease stages during HCC development, the expression of PRELP decreased gradually as disease became more invasive (**Figure 1O**). Generally, PRELP transcription was diminished in HCCs and may be associated with cancer progression.

### Low PRELP expression is associated with poor patient survival

**Figure 2:**
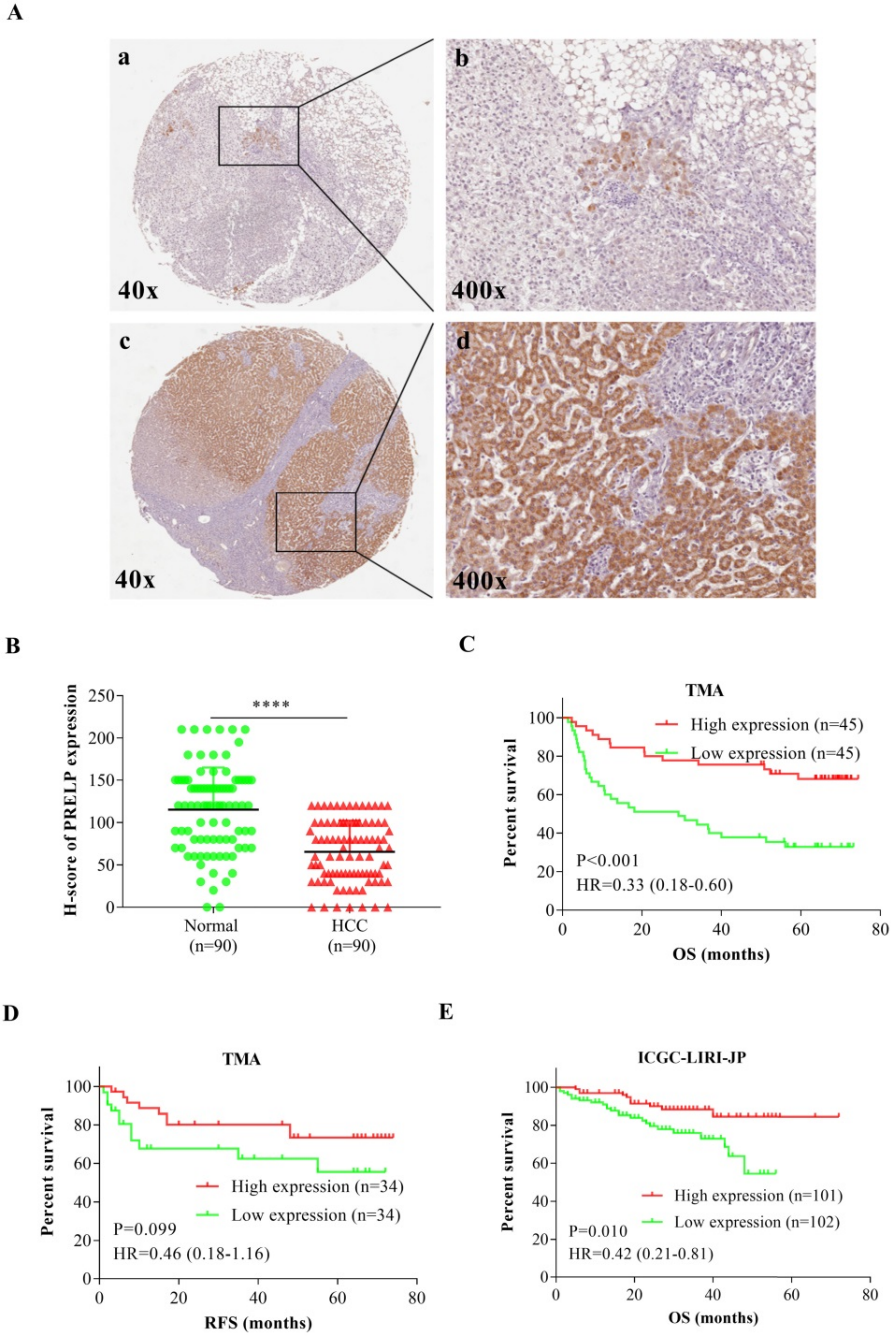
** Down-regulation of PRELP was associated with poor prognoses.** Representative IHC staining images of PRELP in adjacent NTs and HCCs (200 x and 400x magnification) (**A**). The intensity of PRELP expression in IHC staining were evaluated by H-score (**B**) and categorized as low or high protein expression. Patients with low PRELP expression tend to have shorter OS time (**C**), as well as RFS time (**D**), which was confirmed by analysis of ICGC-LIRI-JP (**E**). Abbreviations: IHC: immunohistochemistry; OS: overall survival; RFS: relapse free survival; HR: hazard ratio; ****: *p* < 0.0001; ICGC-LIRI-JP: the Liver Cancer-RIKEN, JP Project from the International Cancer Genome Consortium.

Then we performed IHC staining to analyze PRELP expression on a paraffin-embedded, archived TMA. **Figure 2A** showed that PRELP was located in both cytoplasm and nuclei and its protein expression was dramatically down-regulated in HCC tissues versus controls. The IHC scoring was based on tumor cell staining and each observed tissue component was counted. H-Score plot was graphed to show the quantitative results (*p*<0.0001) (**Figure 2B**). The clinicopathological data was acquired from the TMA including 90 HCC patient cases. All patients were divided into two groups according to high or low PRELP protein expression using the median H-score. The Pearson's χ^2^ test and Fisher's exact test analyses showed that PRELP expression had no significant correlation with age, sex, histological grade, HBV infection, AFP (ug/L), pT stage, pN stage, pM stage, and vessel invasion (**Table 2**). However, cases with low PRELP expression tended to have advanced p-stages (*p*=0.028) and larger tumor size (*p*=0.001). By adjustments of histological grade, tumor size, AFP, p-stage, and vessel invasion, multivariate Cox proportional hazards analyses revealed that overall survival (OS) in HCC patients was independently correlated with PRELP expression [hazard ratio (HR), 0.43; 95% confidence interval (CI), 0.19-0.99; *p*=0.048], along with pstages [HR, 2.95; 95% CI, 1.24-7.02; *p*=0.015] (**Table 3**). Kaplan-Meier survival curves and Log-Rank tests indicated that OS of high PRELP expression cases was longer than that of low PRELP expression with an HR of 0.33 [estimated mean OS, 58.13 months; 95% CI, 50.48-65.78 months; vs. 34.82 months; 95% CI, 26.10-34.54 months; log-rank test, *p*=0.001, **Figure 2C**]. There were 68 patients who provided relapse information during follow-up, the analysis of which demonstrated that there was a trend toward significance that low PRELP expression was correlated with shorter relapse free survival (RFS) time with an HR of 2.17 [estimated mean RFS, 47.34 months; 95%CI, 35.64-59.64 months; vs. 59.66 months; 95%CI, 51.23-68.08 months; log-rank test,* p*=0.099, **Figure 2D**]. To further verify the prognostic value of PRELP, the transcriptomic data from an outside cohort was accessed and analyzed, supporting that patients with low PRELP expression had significantly shorter survival time with an HR of 2.38 [estimated mean OS, 43.62 months; 95% CI, 39.47-47.77 months; vs. 64.39 months; 95% CI, 60.04-68.74 months; log-rank test,* p*=0.010, **Figure 2E**]. To sum up, these findings revealed that low PRELP protein expression in HCCs is associated with poor patient survival.

### Downregulation of PRELP promotes proliferation and migration of cultured HCC cells

**Figure 3:**
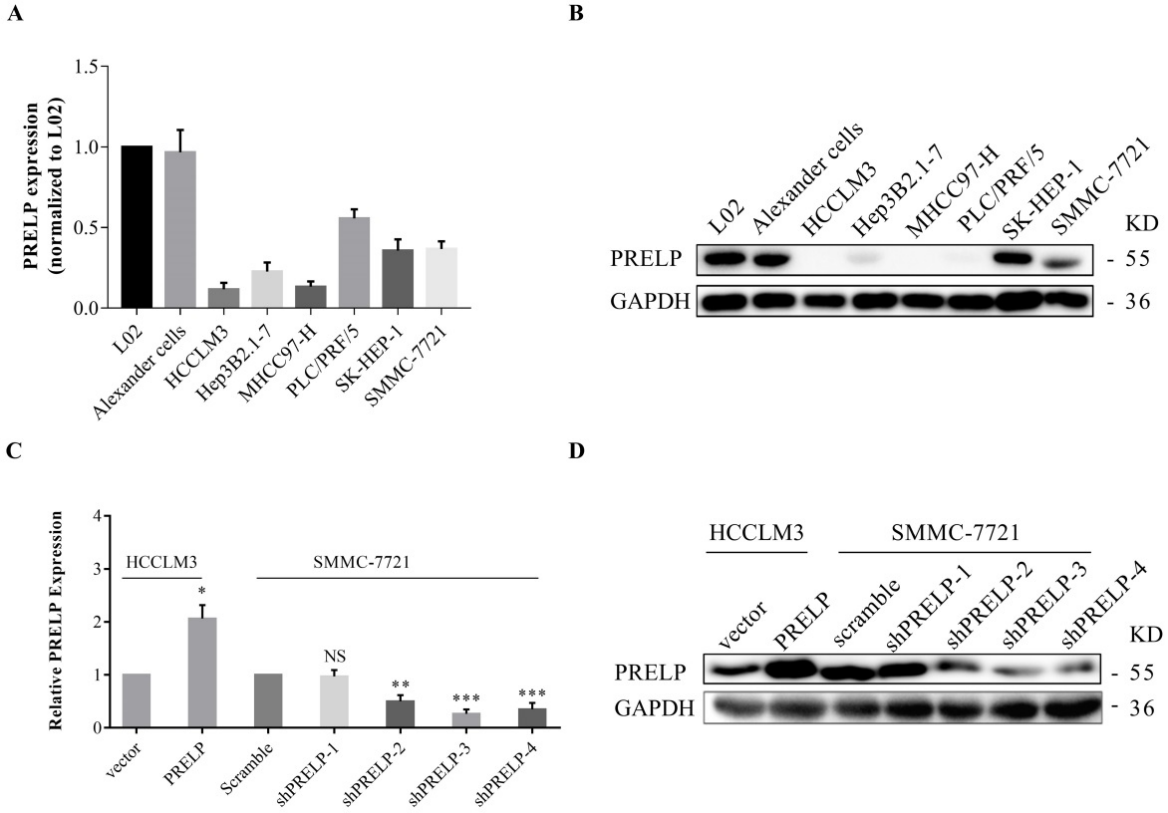
** Endogenous PRELP expression in a normal hepatic cell line and seven HCC cell lines.** PRELP mRNA and protein were respectively examined by qPCR and WB in L02 and 7 HCC cell lines (**A and B**). PRELP expression was normalized by L02 cell line. The mRNA and protein expression of PRELP was respectively determined by qPCR and WB in SMMC-7721and HCCLM3 cells (**C and D**), which were both infected by corresponding lentiviruses. Abbreviations: NS: not significant; *: *p*<0.05; **: *p*<0.01; ***: *p*<0.001; ****: *p*<0.0001 vs. vector control or scramble control; qPCR: quantitative real-time polymerase chain reaction; WB: western-blotting; shRNA: short hairpin RNA.

**Figure 4:**
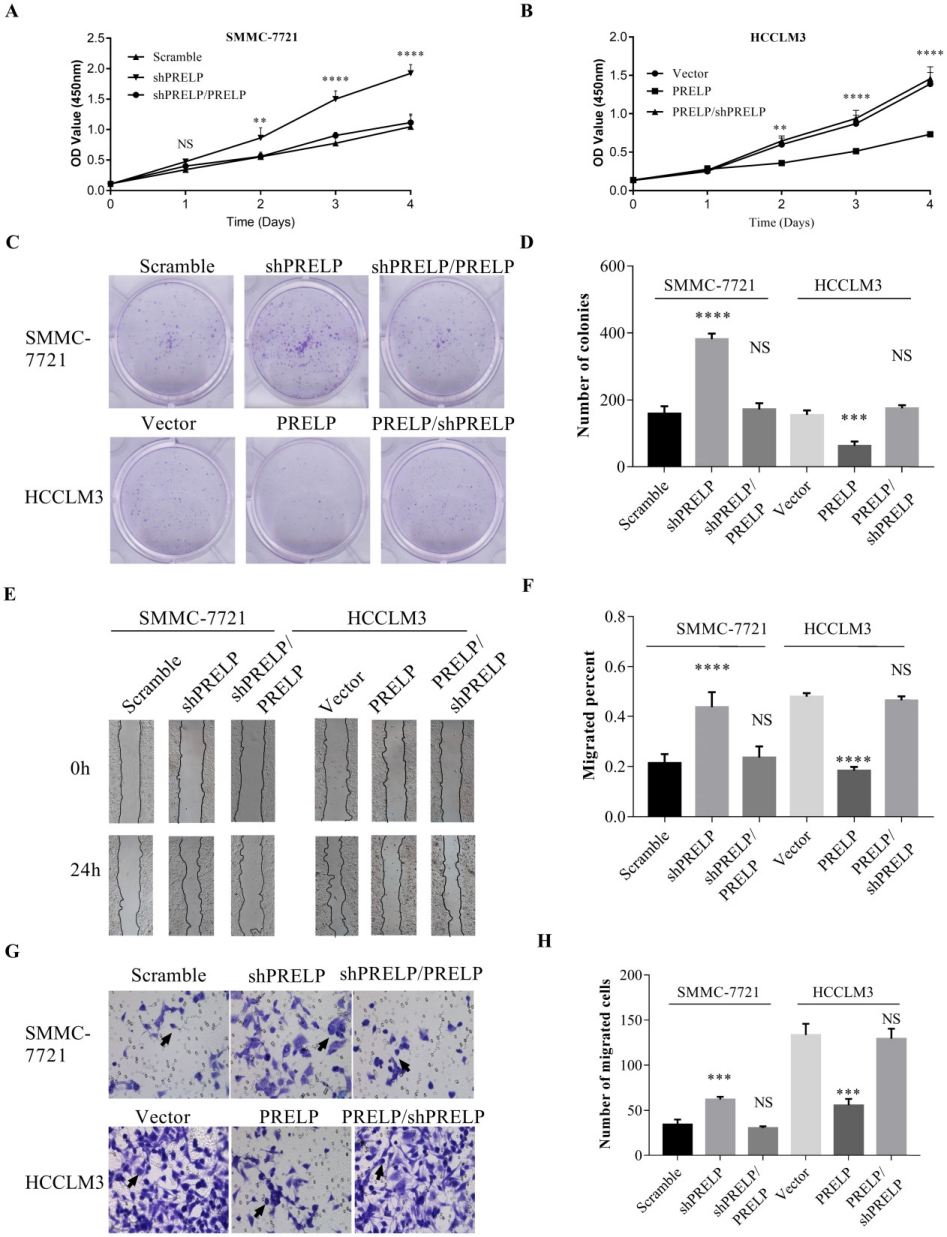
** Overexpression of PRELP inhibited HCC cell proliferation and migration.** CCK8 assay was used to examine proliferation ability of HCC cells (SMMC-7721 and HCCLM3) infected with lentiviruses carrying scramble control or shPRELPs vector control or PRELP (**A and B**). Overexpression of PRELP decreased the mean number of colonies in the colony formation assay while down-regulation of endogenous PRELP increased colony formation ability of HCC cells (**C and D**). Wound healing assay was used to evaluate the migratory ability of these two cells. The representative images (left) and quantitative results(right) of wound healing assay both indicated that overexpressed PRELP inhibited cell migration (**E and F**). The same results of HCC cells were showed by transwell migration assay. Representative micrographs (left) and quantitative results (right) of transwell migration assay both indicated that overexpressed PRELP could limit the migratory ability of HCC cells. Abbreviations: CCK8: Cell counting Kit‑8; NS: not significant; **: *p* < 0.01; ***: *p* < 0.001; ****: *p* < 0.0001 vs. vector control or scramble control; shRNA: short hairpin RNA.

In order to understand the *in vitro* roles of PRELP in HCC cells, we utilized qPCR and WB to evaluate the endogenous expression of PRELP in a hepatic cell line L02 and seven HCC cell lines from the start. As shown in **Figure 3A** and **3B**, PRELP was downregulated in all HCC cell lines except Alexander cells compared to that in L02 cells. Referring to foregoing results, we then ectopically overexpressed PRELP in HCCLM3 cells and knocked down PRELP in SMMC-7721 cells through lentiviruses infection (**Figure 3C** and **3D**). We performed CCK-8 assay in these two cells. Knocking down of PRELP dramatically accelerated the growth of SMMC-7721 cells (**Figure 4A**), by contrast, overexpression of PRELP decelerated that of HCCLM3 cells (**Figure 4B**), compared to respective controls. Similarly, after cultivation for 14 consecutive days, knock down of PRELP markedly increased the mean colony number in the colony formation assay and vice versa (**Figure 4C and 4D**). Next, we performed wound healing assay on these two cell types, and the migratory areas were measured after culturing for 24 hours. The results showed that PRELP knockdown significantly enhanced the migratory capacity of SMMC-7721 cells and PRELP overexpression impeded that in the HCCLM3 cells, in comparison with that of respective control cells (**Figure 4E** and** 4F**). To further validate the roles of PRELP in migration, we then planted aforesaid cell lines into tiny insert chambers, and calculated cell numbers migrating through the chamber after 24h of cultivation. The transwell migration assay further confirmed that PRELP-knockdown cells had enhanced migratory potential than scramble control, while PRELP overexpression cells had diminished migratory potential than vector control (**Figure 4G** and** 4H**). In summary, PRELP may inhibit proliferation and migration of cultured HCC cells.

### PRELP is associated with extracellular matrix (ECM) receptors and cell adhesion pathways

**Figure 5:**
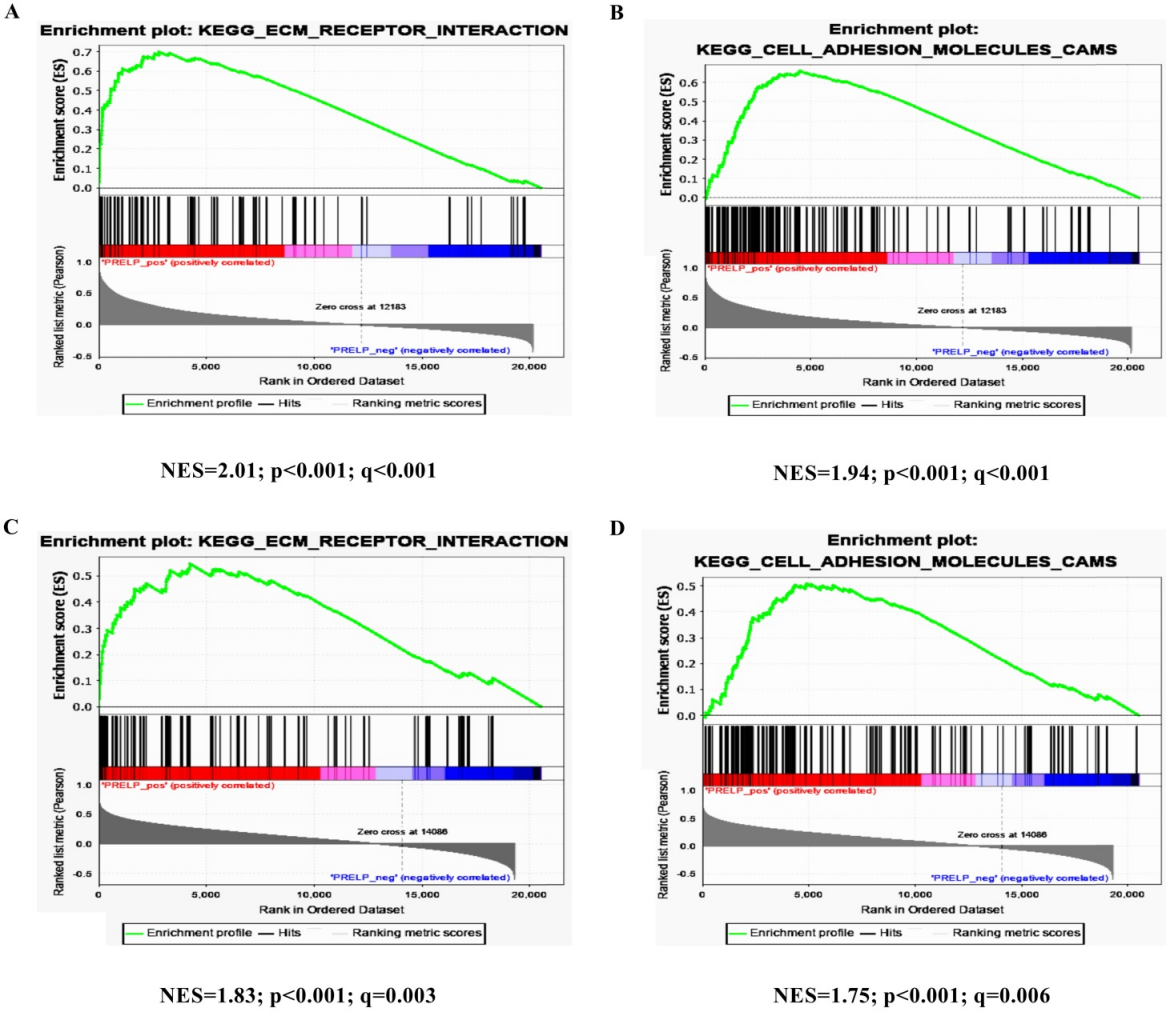
** PRELP was associated with ECM Receptors and cell adhesion pathways.** High PRELP expression was positively associated with cell-adhesion-activated gene signatures (ECM_RECEPTOR_INTERACTION and CELL_ADHESION_ MOLECULER_CAMS) in GSEA of transcriptomic data from HCCs (**A and B**) and adjacent NTs (**C and D**) in TCGA-LIHC. Abbreviations: TCGA-LIHC: The Liver Hepatocellular Carcinoma Project of the Cancer Genome Atlas; ES: enrichment score; FDR: false discovery rate; GSEA: gene set enrichment analysis.

**Figure 6:**
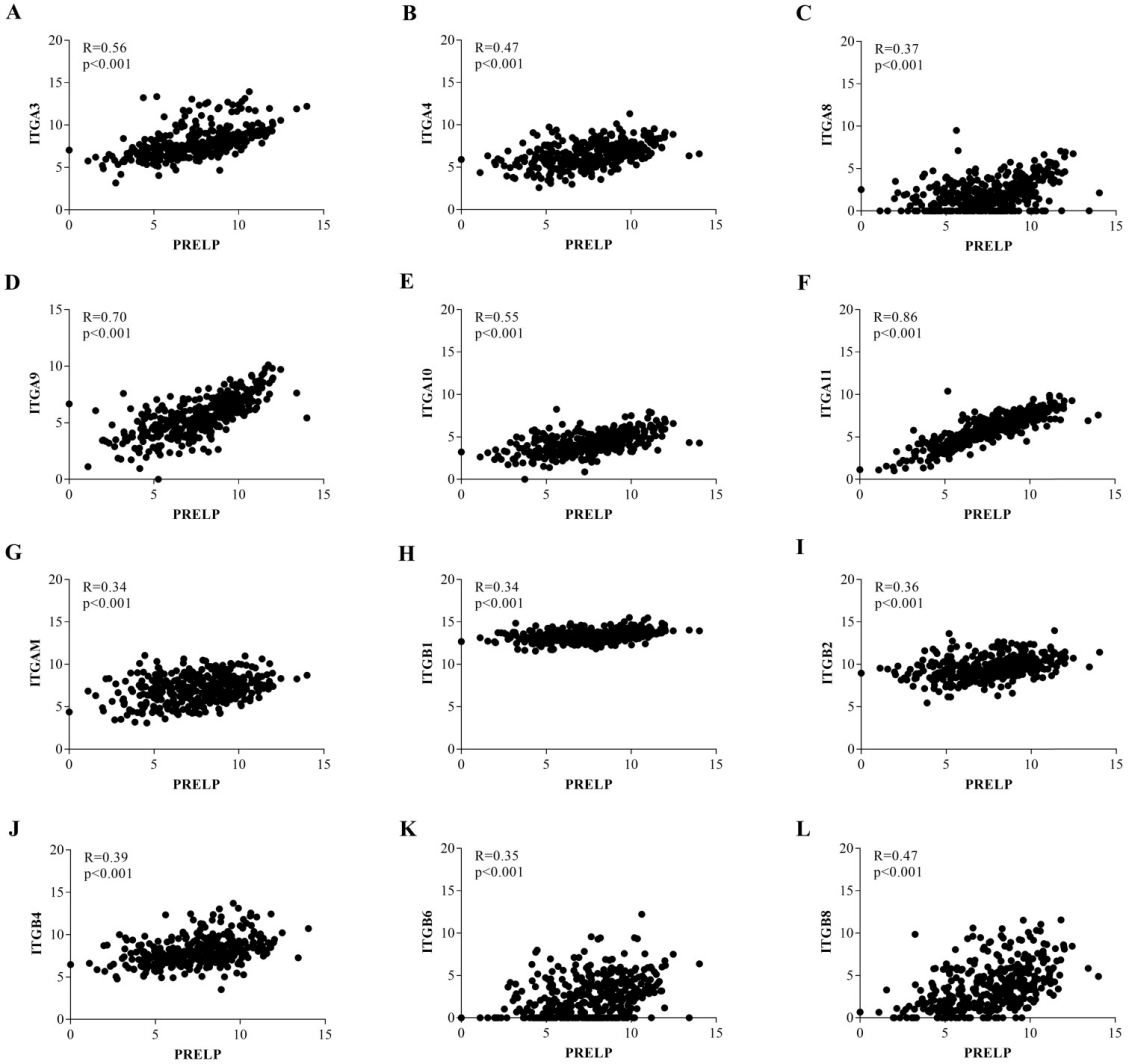
** Expression of PRELP positively correlated with most integrin family members.** Gene-gene correlation analyses between PRELP and selected integrin family members (**A-L**). Abbreviations: ITG: integrin.

To further investigate the potential mechanisms of PRELP in the tumorigenesis and progression of HCC, we performed KEGG pathway analyses on the GSEA platform using the TCGA-LIHC dataset. The analyses of transcriptomic data from both HCCs (**Figure 5A** and** 5B**) and NTs (**Figure 5C** and** 5D**) revealed that high PRELP expression was positively correlated with gene sets of ECM receptors and cell adhesion. Since previous studies suggested that integrin family members mediated cell proliferation, migration and invasion, as well as cross-talks between cells and the ECM 36, while integrins are included in both gene sets mentioned above, we further conducted gene-gene correlation analyses between PRELP and integrin family members using the TCGA-LIHC dataset. The results demonstrated that PRELP expression was significantly and positively correlated with the expression of several integrin family members, including Integrin A3 (ITGA3), ITGA4, ITGA8, ITGA9, ITGA10, ITGA11, ITGAM, ITGB1, ITGB2, ITGB4, ITGB6 and ITGB8 (**Figure 6A-L**). Collectively, these analyses manifested that PRELP may interact with integrins and play critical roles in ECM and cell adhesion of HCC.

## Discussion

Studies for PRELP in human diseases are limited at present, most of which focus on rheumatic diseases. There are a few oncological studies shed light on the roles of PRELP. By glycosylation, PRELP could transform into a 38 kDa core protein which was specifically expressed in chronic lymphocytic leukemia (CLL) cells 37. A previous study based on proteomic identification suggested that high PRELP expression was relevant to better patient prognosis compared to low PRELP expression in resectable PDAC 13. However, Iuga et al. had drew a completely opposite conclusion that high PRELP expression was relevant to poor prognosis of patients in resectable PDAC 38. These seemingly contradictory findings may be due to the influence of different conditions of experiments. In our study, we demonstrated that PRELP was generally down-regulated in HCC tissues in comparison with matched NTs or hepatic cirrhosis. However, analyses of some of the datasets (GSE36376, GSE46444, GSE14323, GSE25097, and GSE6764) presented no significant results, which may be because of their relatively smaller sample sizes and the cohort effect. Besides, PRELP expression status was significantly associated with prognoses of patients. Patients with low expression of PRELP tended to possess shorter OS time and RFS time. Furthermore, patients who had low tissue expression levels of PRELP tended to have larger tumor size and advanced pathological stage. All these show that PRELP is down-regulated in HCCs and has the potential to be a prognostic predictor. Although timely biopsy and tissue fixation had been done after surgical resection, the variant intervals from biopsy to IHC staining may have impact on PRELP expression, thus inevitably bringing bias to the evaluation of prognostic value of PRELP.

Tumor proliferation and metastasis are significant hallmarks of HCC; hence, studies on these aspects would find therapeutic targets to control disease progression 39. It is widely known that continuous activation of multiple pathways leads to uncontrolled cell proliferation and promotes tumor metastasis 40, 41. We found that PRELP knockdown promoted, while PRELP overexpression inhibited cell proliferation and migration. In a word, PRELP may act as a therapeutic target against HCC progression. It has been reported that PRELP could inhibit osteoclastogenesis via impeding transcriptional activity of NF-kB signaling pathway 42, and regulate β-catenin/connexin43 pathway in osteoblast differentiation 7. We assume that the inhibitory role of PRELP in HCC cells may be implemented by modulations of multiple signaling pathways.

ECM, which is mainly comprised of collagens, elastin, fibronectin, laminins, glycoproteins, proteoglycans (PGs) and glycosaminoglycans (GAGs), is an essential and dynamic part of tumor microenvironment 43. PRELP was originally isolated from bovine articular cartilage, which is an essential territorial matrix 44. Electron microscopy showed that PRELP can closely bind to perlecan and procollagen 44, probably because of its special amino-terminal region and the glycosaminoglycan-binding domain 42, 45. There is a study which certified that the amino-terminal region of PRELP increased cell adhesion and regulated their interaction with GAGs 10. Basement membranes (BMs) serve as a net shaped structure of ECM to separate cells from matrix 46. Bengtsson et al. had confirmed that localization of PRELP was adjacent to BMs by confocal immunohistochemistry. These findings verified the collaboration between PRELP and ECM 4. The integrin family, which is wildly reported in HCC, is involved in multiple molecular mechanisms within and outside tumor cells 47. Integrins, which normally bind to ECM, promote cell invasion and metastasis by regulating cell adhesion to basement membrane and altering cell malignant features 47, 48. A previous study reported that activation of ITGB1 signaling dramatically enhanced cell proliferation and invasion in HCC 49. The similar effect has been examined on ITGB3 50, ITGB4 51, ITGB5 52, ITGA1 53, ITGA2 54, ITGA5 55, ITGA6 56, ITGA7 57 in HCC. However, it was worth noting that a recent study found that overexpression of ITGA3 can attenuate the proliferation of HCC cells regulated by LAMB3, and the inhibitory roles have also been reported on ITGA9 in HCC 58. From former studies we made a conjecture that PRELP inhibits HCC progression by interacting with integrin family members within the tumor microenvironment. To confirm it, we performed KEGG pathway analyses on the GSEA platform and the results suggested that high expression of PRELP was positively related to gene sets extracellular matrix receptors and cell adhesion which are main pathways in tumor microenvironment. By further exploration of these two enriched pathways we found that PRELP expression was positively relevant to most integrin family members. Taken together, PRELP may activate extracellular matrix receptors and accelerate cell adhesion by interacting with certain integrin members, then attenuate cell proliferation and invasion. All these observed results need further confirmation both *in vitro* and *in vivo*.

## Conclusion

To sum up, we found that PRELP may serve as a potential prognostic marker and a treatment target against HCC progression. PRELP may cross-talk with integrin-mediated ECM and cell adhesion pathways which require further validation.

## Figures and Tables

**Table 1:** Clinical and pathologic features of HCC patients^*^ (n=90)

Variable	No. of patients (%)
**Sex**	
Male	70 (77.8)
Female	20 (22.2)
**Age**	
≤60	64 (71.1)
>60	26 (28.9)
**Differentiation**	
G1/2	54 (60)
G3	36 (40)
**Tumor size (cm)**	
≤5	47 (52.2)
>5	43 (47.8)
**HBV infection**	
Negative	19 (21.1)
Positive	71 (78.9)
**AFP (μg/L)**	
≤200	45 (50)
>200	45 (50)
**Tumor stage**	
T1	58 (64.5)
T2	17 (18.9)
T3	3 (3.3)
T4	12 (13.3)
**Nodal stage**	
N0	85 (94.4)
N1	5 (5.6)
**M stage**	
M0	88 (97.8)
M1	2 (2.2)
**TNM stage**	
I	56 (62.2)
II	17 (18.9)
III	16 (17.8)
NA	1 (1.1)
**Vessel invasion**	
No	50 (55.6)
Yes	25 (27.8)
NA	15 (16.6)

*Data shown here may be duplicated with those from other published resources that are based on the same cohorts. Abbreviations: NA: information not available HCC: Hepatocellular carcinoma; HBV: hepatic B virus.

**Table 2:** Association between PRELP expression and clinicopathological variables in HCC patients (n=90)

Clinicopathological features	N	PRELP expression		
		Low (45)	High (45)	*P*-value
**Sex**				
Male	70	35 (50.0)	35 (50.0)	
Female	20	10 (50.0)	10 (50.0)	1.000
**Age**				
≤60	64	34 (53.1)	30 (46.9)	
>60	26	11 (42.3)	15 (57.7)	0.486
**Histological grade**				
G1/G2	54	23 (42.6)	31 (57.4)	
G3	36	22 (61.1)	14 (38.9)	0.132
**Tumor size (cm)**				
≤5	47	15 (31.9)	32 (68.1)	
>5	43	30 (69.8)	13 (30.2)	**0.001**
**HBV infection**				
Negative	19	10 (52.6)	9 (47.4)	
Positive	71	35 (49.3)	36 (50.7)	1.000
**AFP (μg/L)**				
≤200	45	18 (40.0)	27 (60.0)	
>200	45	27 (60.0)	18 (40.0)	0.091
**pT stage**				
T1	58	25 (43.1)	33 (56.9)	
T2/T3/T4	32	20 (62.5)	12 (37.5)	0.123
**pN stage**				
N0	85	42 (49.4)	43 (50.6)	
N1-N3	5	4 (80.0)	1 (20.0)	0.616
**pM stage**				
M0	88	43 (49.4)	45 (50.6)	
M1	2	2 (100.0)	0 (0.0)	0.494
pStage^*^				
I	56	23 (41.1)	33 (58.9)	
II/III/IV	33	22 (66.7)	11 (33.3)	**0.028**
**Vessel invasion^#^**				
No	50	22 (44.0)	28 (56.0)	
Yes	25	17 (68.0)	8 (32.0)	0.085

*stages are grouped into I and II/III/IV, ^#^vessel invasion is grouped into No and Yes. Bold type indicates significance. Abbreviations: HCC: Hepatocellular carcinoma; HBV: hepatic B virus.

**Table 3:** Univariate and multivariate Cox proportional hazard models for overall survival in HCC patients (n=90)

Clinicopathological features	Univariate analysis		Multivariate analysis	
	HR [95% CIs]	*P*-value	HR [95% CIs]	*P*-value
**Sex**				
Male	1 [Reference]			
Female	1.47[0.76-2.85]	0.258		
**Age**				
≤60	1 [Reference]			
>60	0.78[0.40-1.51]	0.462		
**Histological grade**				
G1/2	1 [Reference]		1 [Reference]	
G3	2.01[1.11-3.63]	**0.022**	0.94 [0.47-1.89]	0.861
**Tumor size (cm)**				
≤5	1 [Reference]		1 [Reference]	
>5	2.32[1.26-4.27]	**0.007**	1.59[0.72-3.49]	0.250
**HBV infection**				
Negative	1 [Reference]			
Positive	1.06[0.51-2.21]	0.872		
**AFP (μg/L)**				
≤200	1 [Reference]		1 [Reference]	
>200	3.17[1.67-6.00]	**<0.001**	2.35 [1.00-5.55]	0.051
**pStage**				
I	1 [Reference]		1 [Reference]	
II/III/IV	5.11[2.73-9.53]	**<0.001**	2.95 [1.24-7.02]	**0.015**
**Vessel invasion**				
No	1 [Reference]		1 [Reference]	
Yes	4.15[2.12-8.10]	**<0.001**	1.59 [0.73-3.50]	0.246
**PRELP expression**				
Low	1 [Reference]		1 [Reference]	
High	0.33[0.17-0.62]	**0.001**	0.43[0.19-0.99]	**0.048**

Bold type indicates significance. Abbreviations: CI: confidence interval; HR: hazard ratio.

## Abbreviations

HCC: Hepatocellular carcinoma
PRELP: proline/arginine-rich end leucine-rich repeat protein
HCCDB: the Integrative Molecular Database of Hepatocellular Carcinoma
TMA: tissue microarray
GSEA: Gene Set Enrichment Analysis
NTs: normal tissues
HCCs: HCC tissues
OS: overall survival
RFS: relapse free survival
HBV: hepatic B virus
SLRP: small leucine-rich proteoglycan
OA: osteoarthritis
PDAC: pancreatic ductal adenocarcinomas
HPA: The Human Protein Atlas
NX: normalized expression
TCGA-LIHC: the Liver Hepatocellular Carcinoma Project of the Cancer Genome Atlas
ICGC-LIRI-JP: the Liver Cancer-RIKEN, JP Project from the International Cancer Genome Consortium
qPCR: quantitative real-time polymerase chain reaction
WB: western-blotting
shRNA: short hairpin RNA
KEGG: the Kyoto Encyclopedia of Genes and Genomes
ANOVA: analysis of variance
HR: hazard ratio
CI: confidence interval
NS: not significant
GAPDH: Glyceraldehyde 3-phosphate dehydrogenase
CCK-8: Cell Counting Kit-8
CLL: chronic lymphocytic leukemia
PGs: proteoglycans
GAGs: glycosaminoglycans
ECM: extracellular matrix
BMs: Basement membranes
Integrin: ITG
